# American Medical Association

**Published:** 1884-06

**Authors:** 


### Society Proceedings.

### AMERICAN MEDICAL ASSOCIATION.

*Thirty-fifth Annual Meeting, held in Washington, Tuesday, Wednesday, Thursday and Friday, May* 6, 7, 8 *and* 9, 1884.

### TUESDAY—FIRST DAY.

The meeting was called to order in the Congregational Church, at the corner of Tenth and G streets, at 10:30 A.M., by the Chairman of the Committee of Arrangements, Dr. A. Y. P. Garnett. After a prayer by the Rev. William A. Leonard, the Chairman introduced the President of the Association, Dr. Austin Flint, of New York.

Dr. Garnett then delivered the address of welcome, in which he spoke of Washington as a place of eminent and renowned gatherings, where the supreme interests of the whole country are studied and discussed by its largest intellects, in order to the discovery and enforcement of those political and constitutional laws which shall best establish and maintain the well-being of the Republic. It was, he thought, a fit place for the meetings of this body, for no laws devised by human wisdom could be more important to humanity than those laws of life upon which rests the "*salus populi.*" He referred also to the peculiar advantages possessed by this body, which is not affected by sectional interests or by party ambitions. They had no constituencies to whom they were responsible. They moved in a higher plane, and were answerable alone to the dictum of scientific truth. The truths they discovered could not be patented for individual use, or converted into practical profit by huge monopolies. He alluded eloquently to the mission, the duties and responsibilities of the physician, and to his singular experience. What, he asked, in comparison to such experience, was the knowledge of human nature acquired in the confidential council chamber of the lawyer, or learned in the secrecy of the confessional, even when remorse wrings truth from the penitent? Their daily experi ence forced them to recognize with humility that beyond all their knowledge there were experiences they could not understand, laws they could not explain or regulate, and powers and influences they could not control, and should they not, he asked, be taught by the mental, moral and physical relations which these experiences brought before them, an abiding faith in the wisdom of that Being who has made the ministry of pain and death a part of His divine administration in nature and in nature's order of animate life? He referred to the value and importance of these periodical gatherings, and the useful results which have thus far followed the successful administration of the grand organization.

In closing, Dr. Garnett said: "In welcoming you, then, gentlemen, to this beautiful metropolis, I hope I shall be pardoned for the indulgence of that natural pride which prompts me to predict that, at no very distant day, to the many physical beauties and natural advantages which she at present possesses, and which assure her future grandeur, splendor and power, there will be added the sublimest achievements of intellectual effort, the wonderful evolutions and demonstrations of professional science, the highest conceptions and skillful executions of perfected art, representing all the nations of the earth, and conspiring to make Washington the center of those educational and intellectual movements which, in their development, exercise so large an influence in moulding the national character and in shaping the destiny of our people."

The following programme of entertainments was then announced:

*Tuesday*—Reception by the President of the United States, at the Executive Mansion, from 8.30 to 10.30 P. M.; Reception by Mr. and Mrs. L. Z. Leiter, from 9 to 11 P. M.

*Wednesday*—Reception and Entertainment by the Medical Profession of Washington, at the National Rifles' Armory, from 9 to 12 P. M.

*Thursday*—Illumination of the Corcoran Art Gallery, and Reception by Mr. W. W. Corcoran and the Board of Trustees, from 8 to 10 P. M.; Reception by Commissioner and Mrs. Loring, from 9 to 11 P. M.; Reception by Mr. and Mrs. W. T. Hildrup, from 9 to 11 P. M.; Reception by Chief Justice Morrison R. Waite, from 9 to 11 P. M

*Friday*—Reception by the Surgeon-General of the Army and his Staff, at the Army Medical Museum, from 8 to 11 P. M.; Reception by the Hon. George F. Edmunds, President *pro tempore* of the Senate, and the Hon. John G. Carlisle, Speaker of the House of

Representatives, at 8 P. M.; Illumination of the Capitol from 8 to 10 P. M.

COMMUNICATIONS were acknowledged by the Committee of Arrangements from A. Pearce Gould, F. R. C. S. of London, and from other eminent medical men of Europe, and were ordered on the minutes.

THE REGISTRATION.—The Secretary read the list of registered delegates up to the time of the morning session having been called to order, the whole number being five hundred and ninety. On registering, each delegate had subscribed to the following declaration: "In acknowledgment of having adopted the constitution, by-laws and code of ethics of this body, and of my willingness to abide by them, and use my endeavors to carry into effect the objects of this Association, I hereunto affix my name."

INVITED GUESTS.—Dr. J. H. Turnbull, Dr. Jonas A. Marshall, Dr. Gorlet, and the members of the Medical Association of the District of Columbia were invited to seats in the meeting.

A LETTER FROM DR. JOHN L. ATLEE, an ex-president of the Association, expressing his regret at being unable to attend the meeting, was read by the Secretary and ordered on the minutes.

### THE PRESIDENT'S ADDRESS.

The President, Dr Austin Flint, Sr., of New York, then made his address, in which he referred to the early history of the Association and selected as a text for his address the preamble to the plan of organization adopted at the formation of the Association.

With regard to the progress of medicine he said: "No one of those whom I address will deny the assertion that medicine is now progressing as it has progressed in the past, and that it will continue to progress in the future. I do not doubt that the present stage of its progress will hereafter be cited as an important epoch in its history. For the past quarter of a century, histological and clinical studies have tended to develop more and more our knowledge of the existence of specific agents in the causation of diseases. That, as regards certain diseases, these specific agents are micro-organisms, has been demonstrated. The latest discovery in this direction is that of the bacillus tuberculosis, a discovery which is the leading topic in medical literature at the present time. Recent trustworthy researches go far toward demonstrating the existence of specific organisms in pneumonia, typhoid fever, malarial fever, and epidemic cholera; and, reasoning by analogy, it is a logical conclusion that ere long a host of diseases will prove to be parasitic. It is easy to perceive how important must be the bearings of these developments in ætiology and pathology on prophylaxis and therapeutics. A new era is about to be inaugurated in these practical departments of medicine Professor Huxley, in his address at the International Medical Congress in 1881, uttered a prediction in these words: 'It will become possible to introduce into the economy a molecular mechanism, which, like a very cunningly contrived torpedo, will find its way to some particular group of living element, and cause an explosion among them, leaving the rest untouched.' I would rather say that the time will come when means will be found to destroy morbific agents outside of the body, thereby securing the prevention of diseases; and that means will be found to effect the destruction of these agents within the body, thereby arresting the course of diseases. Let us hope that the medical profession in this country will take an honorable part in the labors for the cultivation and advancement of medical knowledge with reference to these most desirable results! At all events, let us continue to recognize and apply medical knowledge, wherever it may originate!"

With regard to the elevation of the standard of medical education, the President said:

"At the present time, the most important of the means for elevating the standard of medical education relate to the preliminary requisites for the study of medicine. The committee appointed in 1846 to report on this subject, recommended that members of the medical profession throughout the United States should satisfy themselves, before receiving young men as students, that they have acquired a 'good education, a knowledge of natural philosophy and the elementary mathematical sciences, including geometry and algebra, and such an acquaintance at least with the Latin and Greek languages as will enable them to appreciate the technical language of medicine and read and write prescriptions' It was also recommended that the medical colleges require a certificate of such an amount of preliminary acquirements before matriculation.

"These requisites are, assuredly, neither in number nor degree, unreasonable. The need of any knowledge of the dead languages has been doubted; but when it is considered that the names of all the organs and tissues in the body, and the nomenclature of diseases, together with terms used in physiology, chemistry, materia medica, pharmacy, surgery and obstetrics, are of either Greek or Latin derivation, it is difficult to understand how the student can dispense altogether with this knowledge. The want of it must, to say the least, be a great drawback. A thorough acquaintance with the language, philologically, or with reference to an appreciation of the literature of Greece and Rome, however, elegant as an accomplishment, is by no means essential as preparatory for the study of medicine, and it may fairly be questioned whether the time usually spent in the study of Greek and Latin authors by those who graduate in arts at our colleges, might not be given with much more profit to physics, chemistry, and the French and German languages. To the requisites recommended by the Association might be added, with advantage, knowledge of the elements of chemistry, leaving only the practical applications of this science in the different departments of medicine to be taught in our medical schools.

It must be confessed that the progress in the matter of the preliminary education of medical students, is less satisfactory than that in medical instruction. Not a few medical students are deficient in the knowledge and the mental training requisite as preparatory for the study of medicine. The fault, exclusive of the students themselves, lies in part with the medical schools, and partly with private preceptors; more with the latter than the former, inasmuch as to them, generally, applications are first made to enter upon the study. I have no wish to be an apologizer for either; there is blame on both sides, and there should be mutual co-operation in an effort to remove the greatest of the present obstacles in the way of elevating the standard of medical education.

The practical question is, what can the Association do to promote more and more the elevation of the standard of medical education? I will meet this question, in the first place, by suggesting what it is desirable should not be done.

It is not judicious to decry medical education in this country as unworthy of any commendation, and as contemptible when contrasted with the educational advantages of other countries. The low estimate in which medical education in America appears to be held by some of our writers, is not only discouraging, but it is without any warrant from facts. Our methods of teaching have certain notable advantages above those of other countries; and in respect of effectiveness in practical branches, we have no occasion to be ashamed if comparisons be fairly made.

It is alike impolitic and unjust to depreciate, as is sometimes done in offensive terms, the medical profession of the United States. As a body, the members of our profession in this country are neither ignorant nor in any respect unworthy. The profession is honorable and honored. In no other country is the social status of its members higher. If a distinction be not always drawn, in public estimation, between true merit and either pretentious ignorance or fraudulent assumption, this is an evil by no means confined to our country; it is incident to the peculiar character of medical knowledge and to human credulity. Granting the need of a better average of professional acquirements, it is not to be effected by abuse of the profession as a whole.

Sweeping charges against medical schools of venality and deception, are not less unwise than unbecoming. As regards their maintenance, the condition of the medical schools in this country, when compared with those of other countries, is anomalous. For the most part they are unendowed, and therefore must be self-sustaining. If they cannot offer adequate inducements for patronage, they cannot exist. This condition has its advantages, but also its drawbacks. There is but little ground for the accusation of resorting to dishonorable methods to secure patronage. I may venture to assume some degree of competency to speak with confidence in regard to this matter, it having fallen to my lot to have been connected with several medical colleges, and I aver my belief that the endeavor to entice students by any expe tation of easy graduation, or by any other disreputable inducements, must be rare exceptions to the rule. Granting that there are exceptions, and that some colleges may be guilty of flagrant abuses of their chartered rights, is it not more just, and in all respects better, to pursue measures for the extinction of their rights, as for rendering their degrees nugatory, than to make the evil doings of a few the ground for indiscriminate abuse, embracing colleges which are striving to do their duty to their pupils, to the medical profession and to the public?

It is injurious and an injustice to assert, as is often done, that a large proportion of those pursuing the study of medicine in this country, are governed by a desire to gain admission to the profession as quickly, as cheaply, and with as little medical knowledge as will suffice for that object. Here it may not be too presuming in me to speak with some authority, having, as a teacher for forty years, been brought into relations with many thousand medical students from the Northern, Southern, Eastern and Western States of the Union. As a class, they will compare favorably with those preparing for the sister profession or for any of the callings of life.

A large proportion evince a degree of diligence and perseverance not excelled, as I believe, in any other country. I rejoice in the present opportunity to bear this testimony, based on personal knowledge, in behalf of the much and undeservedly abused American medical student.

Elevation of the standard of medical education is not to be promoted by means such as have just been adverted to. Vituperation does not supply motives for improvement. Reforms are not favored by obloquy. He who is content to do battle with the sword of ridicule, has not the spirit of a true reformer. I have no disposition to deny or to take the attitude of an apologist for defects and abuses which affect, directly or indirectly, the standard of medical education. Some of the evils most complained of might be obviated by the united action of the members of the medical profession. Much is said respecting the overcrowding of the profession by the large number of annual graduates, and this evil is attributed to the number of medical colleges. Assuming for the nonce, the fact, together with the explanation, and that the evils overbalance the advantages of an active competition among the colleges, how is the number of these to be reduced? Reliance on State legislators are likely to take away charters which have been granted, and they will probably continue to be complaisant enough to grant new charters to persistent applicants. But suppose all private preceptors were to induce their pupils not to connect themselves with colleges which are either misplaced or deficient in the means of instruction; a certain number would cease to exist, and the result would exemplify the Darwinian doctrine of the "survival of the fittest."

With regard to the Code of Ethics he spoke at length, quoting from several of the ex-presidents to prove the good influence it had exerted in the past and what it promised in the future. In closing this part of his address, he said:

"Every member of our profession is often made painfully aware, not only of apathy but misapprehension, in the public mind, as regards our duties, responsibilities and requirements. The popular diffusion of our Code of Ethics, allowing it to speak for itself, would go far toward the removal of many errors in regard to our profession. A valuable service, not less to the public than to the profession, might be rendered by proper efforts in this direction. In this way the public may perhaps be led to understand and appreciate the rules of etiquette for which we are often held up to ridicule; our opposition to secret nostrums, and patented discoveries and improvements, and our refusal of fellowship with irregular practitioners. Recurring to the last named topic, many suppose that fellowship with those who are not of the regular profession is refused either from a false sense of dignity or from prejudice, regardless of the dictates of humanity. Now, the ethical code neither in letter nor spirit inculcates nothing in conflict with the humane exercise of our calling. This is a superfluous assertion to those familiar with the teachings of the Code. The principle of humanity underlies all its practical precepts. Quoting words which have been already quoted, "The good of the patient is the sole object in view." There are circumstances under which the higher law of philanthropy, placing in abeyance those rules of the ethical code, as well as of etiquette, which under other circumstances claim obedience, not only permits but commands members of the regular profession to bestow their professional services without stopping to ask whether a practitioner with whom chance has brought them into association be, or be not, a "fit associate in consultation." Under such circumstances, association with an irregular practitioner, governed by a supreme regard for humanity, involves neither fellowship nor professional application, and it is to these alone that ethical restrictions apply. Let it not be conceded for an instant that there can be any antagonism between humanity and medical ethics. Nor is it difficult in particular instances to decide, practically, in what way the dictates of humanity are to be harmonized with ethical rules. Regarded in the light derived from both these sources, the path of duty is sufficiently plain. Any act honestly performed purely for a humane purpose, cannot but be consistent with the principle which underlies our admirable Code of Ethics. But it is to be added, professional association with those who, in the language of the Code, are not "fit associates in consultations" except in emergencies when philanthropy, for the time being, abolishes all restrictions, contravenes the humane objects of our profession just so far as the useful applications of legitimate medicine are thereby trammeled or curtailed. If it be inhuman under certain circumstances to decline association with irregular practitioners, it would be equally or more so, under other circumstances, to make those concessions which professional fellowship requires to practitioners who profess hostility to medical science and to the regular medical profession. The refusal of fellowship and affiliation with irregular practitioners, thus, is sanctioned, not only by a sentiment of self-respect, and respect for our profession, but by a proper regard for the dictates of humanity."

The President recommended that steps be taken to have the International Medical Congress meet in this country in 1887. In regard to this recommendation he said: "My suggestion in regard to the International Congress is not made solely on my own responsibility, but at the instance of several well known for their active interest in the welfare of the American medical profession. By one of the most eminent of these, I was requested, quite recently, to make the suggestion, in a letter written with a hand tremulous from the serious illness which has deprived the Association of his presence at this meeting; one whose absence is a disappointment to many who were desirous to behold once more the genial, benignant countenance which has hitherto greeted us at our annual meetings; one whose writings have made American surgery illustrious, who is revered by the many thousand practitioners who have listened to his instructions, and who is honored, not only in our country, but in other countries, as no member of the medical profession in America was ever before honored; one admired by all who have known him, and beloved by those who have enjoyed the privilege of his friendship. I know that I speak the heartfelt sentiment of every one who hears me, and of our brethren throughout the whole country, when I address a fervent wish that Prof. Samuel D. Gross might be spared yet many years to his family, his friends, and the profession which he adorns."

At the close of the President's address Dr. Toner, of Washington, moved that a vote of thanks be tendered the President for his able address, and that it be requested for publication. Adopted.

By Dr. Toner:

WHEREAS, It has come to the knowledge of the American Medical Association that one of its former presidents, a surgeon of world-wide reputation, whom we miss at this meeting, is confined to his home by serious illness; therefore be it

*Resolved*, That the American Medical Association tenders Professor Gross its heartfelt sympathy in his sufferings, and expresses its sincere hope for his speedy recovery, and for many years of continued usefulness in the profession he has so signally adorned.

Adopted.

Dr. Gihon, of the navy, moved that that portion of the President's address referring to Professor Gross be telegraphed to him at once as an appropriate form of expression of the sentiments of the Association. Carried.

Dr. Richardson stated that he had only recently come from the bedside of Dr. Gross, who, when asked whether he had any word to send the Association, said, in a feeble voice, "Give them my love."

On motion of Dr. Sayre, the resolutions offered by Dr. Toner were ordered to be telegraphed to Dr. Gross. And it was further resolved that the expression of love on the part of Dr. Gross toward the Association be acknowledged and reciprocated.

The Secretary read a telegram from Dr. Baldwin, of Alabama, regretting his inability, on account of illness, to be present at the meeting.

Dr. Sayre moved that that portion of the President's address requesting that the International Medical Congress meet in this country in 1887 be referred to a special committee of five, of which Dr. Billings should be the chairman. Carried.

MEDICAL ETHICS.—Several resolutions were offered regarding that portion of the President's address relating to medical ethics, for which the following, by Dr. Ferguson, of New York, was finally substituted:

*Resolved*, That that portion of the President's address referring to the subject of medical ethics be referred to a committee of seven, to report upon the recommendations therein contained as speedily as possible. Adopted.

The Association then adjourned until Wednesday morning, at 10 o'clock.

### WEDNESDAY—SECOND DAY.

The meeting was called to order at 10 A. M., by the President. Prayer was offered by the Rev. W. A. Bartlett, D.D.

The President announced the death of Professor Samuel D. Gross, of Philadelphia, and appointed the following committee to take such action as it might think proper in the matter: Dr. Richardson, of Louisiana; Dr. Sayre, of New York; Dr. Packard, of Pennsylvania; Dr. Hamilton, of New York; Dr. Gunn, of Illinois; Dr. Briggs, of Tennessee; and Dr. Hays, of Pennsylvania. On motion, the President of the Association was added to the committee as its Chairman. The Secretary read a dispatch from Dr. Samuel W. Gross, thanking the Association for its telegraphic message expressing sympathy, but stating that it had not been received until after his father's death. Dr. Gross's dispatch was ordered on the minutes.

The President appointed the following general committee on his annual address: Dr. N. S. Davis, Dr. W. W. Dawson, Dr. W. T. Briggs, Dr. T. S. Prout, Dr. Starmont and Dr. H. B. Ransom. As the special committee, he named the following gentlemen: Dr. J. S. Billings, Dr. L. A. Sayre, Dr. R. H. Fitz, Dr. I. M. Hays and Dr. H. F. Campbell.

The committee appointed to frame resolutions in favor of securing more competent medical and sanitary service on board trans-oceanic passenger vessels reported, through its chairman. Dr. A. N. Bell, of New York, that a bill had been prepared and presented to Congress which covered the matter. Dr. Bell went on to state that the mortality on board emigrant ships entering the port of New York for four years, up to 1883, was 35.12 in a thousand, being considerably greater than for a like period ending in 1873. This death-rate was at least three times as great as it ought to be. The causes of death were largely negligence and filth.

DR. KEYSER, of Pennsylvania, said the bill before Congress was excellent, so far as it went, but it did not go far enough; it provided that the ship's surgeon might report to the captain, and the captain to the company—but to whom was the company to report? The bill should require the surgeon to report directly to the United States Government.

DR. IRWIN, an English physician, thought the object of the bill was excellent, but that it was impracticable as it stood, and would prove ineffectual. He suggested certain points of improvement.

On motion, the report was received, and the committee continued, the suggestion having been made that information in regard to the matter be reduced to writing and submitted to the committee.

By Dr. Pratt, of Michigan:

*Resolved*, That the American Medical Association, now in session, urge upon Congress the necessity of suitable and efficient legislation to promote the well-being of immigrants to this country, and to protect our public health.

Adopted.

Dr. J. V. Shoemaker, of Philadelphia, chairman of section of Practice of Medicine, read his address.

Dr. F. A. Reamy, of Cincinnati, chairman of section on Obstetrics, read his address, which consisted of notes of 231 cases of laceration of the cervix uteri, in which he had operated.

VIVISECTION.

Dr. Henry Smith, of Pennsylvania, then offered the following, which, after being warmly discussed by Dr. Keyser, of Philadelphia, and Dr. J. C. Dalton, of New York, were adopted with but one dissenting voice:

WHEREAS, It appears that an effort is being made to restrict by legislative action the practice of investigation in medical science by experiments on animals; and

WHEREAS, In the opinion of this Association, such restriction is not needed for the guidance of medical men in their investigations, and would be an injury and a hindrance to the pursuit of medical knowledge and the improvement of the medical art; therefore,

*Resolved*, That a standing committee of seven, with power to increase its number, be appointed by the President of the Association, to be known as the "Committee on Experimental Medicine of the American Medical Association," charged with the duty of opposing, by all legitimate means, any interference with the progress of medical science by unwise or illegitimate legislation.

The President appointed the following gentlemen as said committee: Dr. William Pepper and Dr. James Tyson, Pennsylvania; Dr. Judson, of Maryland; Dr. J. C. Dalton and Dr. Austin Flint, Jr., of New York; and Dr. John S. Billings, of the army.

THURSDAY—THIRD DAY.

The meeting was called to order by the President at 10 A. M., and prayer was offered by the Rev. W. Paret.

Vacancies in the Board of Trustees were filled by appointment by the President as follows: Dr. E. D. Ferguson, of New York; Dr. W. T. Briggs, of Tennessee; Dr. J. E. Reeves, of West Virginia; Dr. J. W. Prewitt and Dr. George Peck, of the navy; and Dr. D. A. Starmont, of Kansas.

THE ARMY MUSEUM AND LIBRARY.—The committee appointed to urge the provision of commodious and fire-proof buildings for the Army Medical Museum and the Library of the Surgeon-General's office, reported that a memorial to Congress recommending that $5,000 be appropriated for the improvement of the museum had not been acted upon in the committee of Congress; but that the committee had reported favorably to an appropriation of $10,000 for the library. The report was accepted, and the committee continued.

GOVERNMENT PROVISION FOR SCIENTIFIC RESEARCH.—Dr. George M. Sternberg offered the following resolutions:

*Resolved*, That we earnestly petition the Congress of the United States to make suitable appropriations for the prosecution of scientific researches relating to the causes and prevention of the infectious diseases of the human race, to be expended under the direction of the National Board of Health; and that a permanent detail of one medical officer of the army and one of the navy be authorized for the prosecution of researches of this nature.

*Resolved*, That a committee of five members of this Association be appointed to present copies of these resolutions to the Speaker of the House of Representatives, to the President of the Senate, and to the chairmen of the Committees on Public Health of the House and of the Senate.

The resolutions were adopted, and the President appointed the following named gentlemen to constitute the committee: Dr. Sternberg (chairman), Dr. Albert L. Gihon, Dr. I. M. Hays, Dr. J. C. Dalton, and Dr. J. E. Reeves.

THE REPORT OF THE BOARD OF TRUSTEES.—The chairman, Dr. J. M. Toner, of Washington, read a report. The editor's report included in the report of the Board of Trustees was read by Dr. Davis. It stated that the actual circulation of the *Journal* had been 3,436, of which 3,271 were among members and subscribers. The total income of the *Journal* had been $18,547.50. It was thought that $500 balance would remain after all expenses were paid. The report was adopted.

The Committee on Nominations, through its chairman, Dr. Hooper, made the following report:

President, Henry F. Campbell, of Georgia; First Vice-President, J. S. Lynch, of Maryland; Second Vice-President, S. D. Mercer, of Nebraska; Third Vice-President, J. W. Parsons, of New Hampshire; Fourth Vice-President, H. C. Ghent, of Texas. The next meeting will be held in New Orleans, beginning the last Tuesday in April, 1885. Members of the Judicial Council: J. K. Bartlett, of Wisconsin; J. H. Murphy, of Minnesota; J. M. Toner, of the District of Columbia; William Brodie, of Michigan; H. D. Holton, of Vermont; A. B. Sloan, of Missouri; —— Ulrich, of Pennsylvania; and W. M. Beach, of Ohio. Secretary, W. B. Atkinson, of Pennsylvania; Assistant Secretary, W. H. Watkins, of Louisiana; Treasurer, R. J. Dunglison, of Pennsylvania; Librarian, —— Kleinsmidt, of the District of Columbia. Chairmen and Secretaries of Sections: Medicine, H. D. Didama, New York, G. M. Garland, Massachusetts; Obstetrics, R. S. Sutton, Pa., J. T. Jelks, Arkansas; Ophthalmology and Otology, J. A. Whil, Virginia, Eugene Smith, Michigan; Surgery and Anatomy, Duncan Eve, Tennessee, C. B. King, Pennsylvania; Diseases of Children, J. L. Pape, Texas, S. S. Adams, D C.; State Medicine, E. W. Schauffler, Kansas, J. N. McCormick, Kentucky; Oral and Dental Surgery, A. W. Harlan, Illinois, J. E. Mears, Pennsylvania. Trustees of the Journal: H. F. Campbell, Georgia; J. H. Packard, Pennsylvania, L. Connor, Michigan. Chairman of the Committee on Necrology, J. M. Toner, D. C. Chairman of the Committee on State Medicine, J. A. Dibrell, sr., Arkansas.

The address on surgery was read by title only.

### FRIDAY—FOURTH DAY.

The meeting was called to order by the President at 9:30 A. M.

VIVISECTION.—Dr. Dalton, chairman of the committee regarding experimentation on animals, offered the following:

*Resolved*, That this Association desires to express its earnest conviction that experimentation on animals is most useful to promote medical science, and can be intrusted only to members of the medical profession.

*Resolved*, That the committee be continued. Carried.

THE BOARD OF TRUSTEES.—A question arose whether nominations to fill vacancies in the Board of Trustees should be filled by the special committee on the trustees or by the general nominating committee.

Dr. Ferguson, chairman of the special committee, offered a resolution to the effect that the committee approved of the nominations which had been made by the nominating committee—namely: Dr. Campbell, Dr. Packard, and Dr. Connor. Carried.

Dr Grissom made a motion declaring it to be the sense of the meeting that the trustees should be nominated by the nominating committee. Carried.

The committee on the President's address reported, through Dr. Davis, its chairman, that no explanation regarding the code should be made without deliberation. Dr. Davis personally offered the following:

*Whereas*, Persistent misrepresentations have been and are being made concerning certain provisions of the Code of Ethics,

*Resolved*, That the President appoint a committee of five permanent members, to report at the next meeting of the Association such explanatory declarations on the subject as the committee may deem proper. Carried.

The Committee on Nominations changed the officers of the section in Oral and Dental Surgery to W. W. Allport, President, and E. C. Briggs, Secretary.

The treasurer's report showed a balance of $2,212.00.

On motion, the annual dues were continued at $5.00.

The President elect, Dr. Henry F. Campbell, of Georgia, was then introduced by the President and addressed the Association in a few timely remarks.

Dr. Brodie moved a vote of thanks in general to the people of Washington, after which the President, in a few appropriate remarks, adjourned the Association.

The next meeting will be held in New Orleans, beginning the last Tuesday in April, 1885.

The following Georgians were in attendance: Drs. Henry F. Campbell and Eugene Foster, Augusta; F. O. Powell, Milledgeville; Wm. F. Holt, H. McHatton, Macon; C. D. Smith, Newnan; W. B. Wells, Red Clay; Robt. Battey, Rome; A. G. Whitehead, Waynesboro; E. K. Bozeman, J. A. Logan, S. B. Hawkins, Americus; B. R. Doston, Blakeley; W. F. Westmoreland, H. V. M. Miller, J. F. Alexander, E. L. Connally, D. H. Howell, and James A. Gray, Atlanta.

